# Sepsis as an independent risk factor in atrial fibrillation and cardioembolic stroke

**DOI:** 10.3389/fendo.2023.1056274

**Published:** 2023-01-30

**Authors:** Yiming Leng, Yalan Li, Jie Wang, Peizhi Deng, Wei Wang, Jingjing Wu, Wenjuan Wang, Chunyan Weng

**Affiliations:** ^1^ Department of Cardiology, The Third Xiangya Hospital, Central South University, Changsha, China; ^2^ Clinical Research Center, The Third Xiangya Hospital, Central South University, Changsha, China

**Keywords:** stroke, atrial fibrillation, sepsis, electrolyte disorder, cardiogenic diseases, causal effect, meta-analysis

## Abstract

**Background:**

Electrolyte balance is an important factor to sustain the homeostasis of human body environment and in sepsis pathogenesis. Many current cohort-based studies have already revealed that electrolyte disorder may intensify sepsis and induce stroke. However, the corresponding randomized controlled trials did not show that electrolyte disorder in sepsis has a harmful effect on stroke.

**Objectives:**

The aim of this study was to examine the association of genetically sepsis-derived electrolyte disorder with stroke risk using meta-analysis and Mendelian randomization.

**Results:**

In four studies (182,980 patients), electrolyte disorders were compared with stroke incidence in patients with sepsis. The pooled odds ratio (OR) of stroke is 1.79 [95% confidence interval (CI): 1.23–3.06; *p* < 0.05], which shows a significant association between electrolyte disorder and stroke in sepsis patients. Furthermore, in order to evaluate the causal association between stroke risk and sepsis-derived electrolyte disorder, a two-sample Mendelian randomization (MR) study was conducted. The genetic variants extracted from a genome-wide association study (GWAS) of exposure data that are strongly associated with frequently used sepsis were used as instrumental variables (IVs). Based on the IVs’ corresponding effect estimates, we estimated overall stroke risk, cardioembolic stroke risk, and stroke induced by large/small vessels from a GWAS meta-analysis with 10,307 cases and 19,326 controls. As a final step to verify the preliminary MR results, we performed sensitivity analysis using multiple types of Mendelian randomization analysis.

**Conclusion:**

Our study revealed the association between electrolyte disorder and stroke in sepsis patients, and the correlation between genetic susceptibility to sepsis and increased risk of cardioembolic stroke, hinting that cardiogenic diseases and accompanying electrolyte disorder interference in due course could help sepsis patients get more benefits in stroke prevention.

## Introduction

Over the past three decades, the annual number of deaths due to stroke has increased dramatically, which became the second leading cause of death and the third leading cause of disability according to the World Health Organization. Stroke cases were reported in 2019 at 12.2 million incident cases and 101 million prevalent cases worldwide, which increased respectively by 70% and 85%, in contrast to 1990. Emphasizing primary prevention strategies and reducing exposure to stroke risk factors were crucial to addressing the global stroke burden (1). A growing body of research showed that cardiovascular/metabolic disorders and poor living conditions such as hypertension, obesity, smoking, and alcoholism have become important risk factors for atrial fibrillation (AF) and stroke (1, 2). Moreover, it is worth noting that new-onset AF in the setting of severe sepsis appears associated with an increased risk of stroke and hospital mortality (3). Sepsis is now characterized as a life-threatening organ dysfunction caused by a dysregulated host response to infection (4, 5). Arterial hypotension and low blood volume is the most common feature of vascular dysfunction among sepsis patients, which could cause disseminated intravascular coagulation (DIC) along with coagulopathy, resulting in severe cerebrovascular events (6). Infection prevention and control appear to be particularly important for reducing hospitalization and mortality among stroke patients. Evidence has demonstrated that the risk of stroke is high after sepsis and will persist for up to a year. Younger sepsis patients have a particularly increased risk of stroke after sepsis (7). Multiple cohort studies suggest that a history of sepsis is positively correlated with the incidence of stroke and shows similarity to our hypothesis that patients with stroke-induced electrolyte disorder will get more benefits of infection prevention efficiently (8–10). It remains unclear, however, whether the effect of sepsis on stroke incidence in observational studies can be attributed to confounder bias such as subjects who have lower income or unhealthy lifestyles. Confounders like these may affect cerebrovascular condition to some extent. Reverse causation between sepsis and stroke should also be taken into account additionally.

By utilizing genetic variants as instrumental variables to assess the impact of genetic variants, we improve the design through meta-analysis and Mendelian randomization (MR) analysis as a means of overcoming the bias and set up a robust causal inference between sepsis and risk of cardioembolic stroke and large/small vessel disease without involving confounders and reverse causations (11).

## Methods

### Search strategy

A comprehensive search of electronic databases, including PubMed, Embase, and Cochrane Library, was performed for articles investigating AF or stroke in patients with sepsis or electrolyte disorder up to 1 August 2022. A number of search terms were used, including “atrial fibrillation” and “ electrolyte disorder” or “sepsis”. We conducted the search with the assistance of two independent investigators (YL and SP). Neither study design nor language restriction was imposed in this meta-analysis, but only English-language articles were included.

### Inclusion and exclusion criteria

In a preliminary screening, the title and abstract of the studies were reviewed to determine their relevance to AF in sepsis and electrolyte disorders. A thorough review of the articles was conducted to identify studies that conform to our inclusion criteria: (a) studies of patients exposed to AF during sepsis, including electrolyte disorder, septic shock, or severe sepsis (including the management of sepsis); (b) in the control group, patients without AF were present with septic shock or sepsis, but had electrolyte problems; and (c) the study included original data on outcomes of concern (length of stay in intensive care units (ICUs) and hospitals, and in-hospital/post-discharge mortality, as well as the likelihood of recurrence of AF or stroke). Those studies that met the following exclusion criteria were excluded from the study: (a) reviews, editorials, letters, case reports, system reviews, or meta-analyses; (b) studies that were conducted in patients with a history of AF (as opposed to patients experiencing AF for the first time); and (c) ORs of each outcome and 95% confidence intervals (CIs) cannot be estimated because of insufficient data. Even though there were differences between studies when it came to how severe sepsis was defined, most studies identified it as sepsis and accompanying multiple organ dysfunction.

### Data extraction and quality assessment

Investigators from two different institutions evaluated all candidate studies (YL and SY) independently. The following items were recorded for each study: the last name of the primary author, the year of publication, the study design, the number of cases of sepsis, the average age of the study population, the percentage of different genders, diagnostic information on AF, and outgoing events. The Newcastle–Ottawa scale (NOS) was used in the quality assessment of each candidate study conducted by two independent investigators (YL and SP) (12). Study quality is determined by three facets: selection (0 to 4 points), comparability (0 to 2 points), and outcome assessment (0 to 3 points). Studies with a score of 6 or higher are considered high quality.

### Instrumental variable selection

Genetic instruments for “sepsis” were identified and extracted from the databases of UK Biobank and HUNT study, with a statistically significant threshold (*p* < 5 × 10^−6^, *r* ^2^ < 0.001). Sepsis was a binary variable, defined by the International Classification of Diseases, 9th and 10th versions. The study included 462,918 subjects, with 10,154 cases and 454,764 controls. *F* statistic was used to demonstrate the strength of the relationship between SNPs and exposures. *F* statistic related to the explained variance for exposure (*R* ^2^), sample size (*n*), and number of SNPs (*k*) by the formula *F* = [(*n* − *k* − 1)/*k*]/[*R* ^2^/(1 − *R* ^2^)]. Generally, robust sepsis prediction by selected SNPs can be indicated through *F* > 10 (PMID: 21414999).

### Outcome data sources

GWAS summary data of ischemic stroke (case/control = 10,307/19,326; overall = 29,633) was extracted from a meta-analysis of 12 individual GWAS. To further explore the etiology of ischemic stroke, we also extract the summary outcome data for different subtypes including cardioembolic stroke, large vessel disease, and small vessel disease.

### Statistical analysis

The original data explored from the candidate articles were used to estimate unadjusted ORs and 95% CIs of mortality, stroke, and recurrence of AF. Studies with adjusted ORs were used when reported in the articles. Heterogeneity was assessed through Cochran’s *Q* test and Higgins’ *I* ^2^ statistic among the studies, and *p*_h_ < 0.12 or *I* ^2^ > 50% showed significance (13). Significant heterogeneities, if any, were determined by using a random-effects model or a fixed-effects model. We conducted subgroup analyses, sensitivity analyses, and meta-regressions to investigate and explain heterogeneity between studies. A *p*-value of less than 0.05 is considered statistically significant, whereas Begg’s and Egger’s tests are used to test for publication bias. STATA version 16 was used for all statistical analyses.

**Figure 1:**
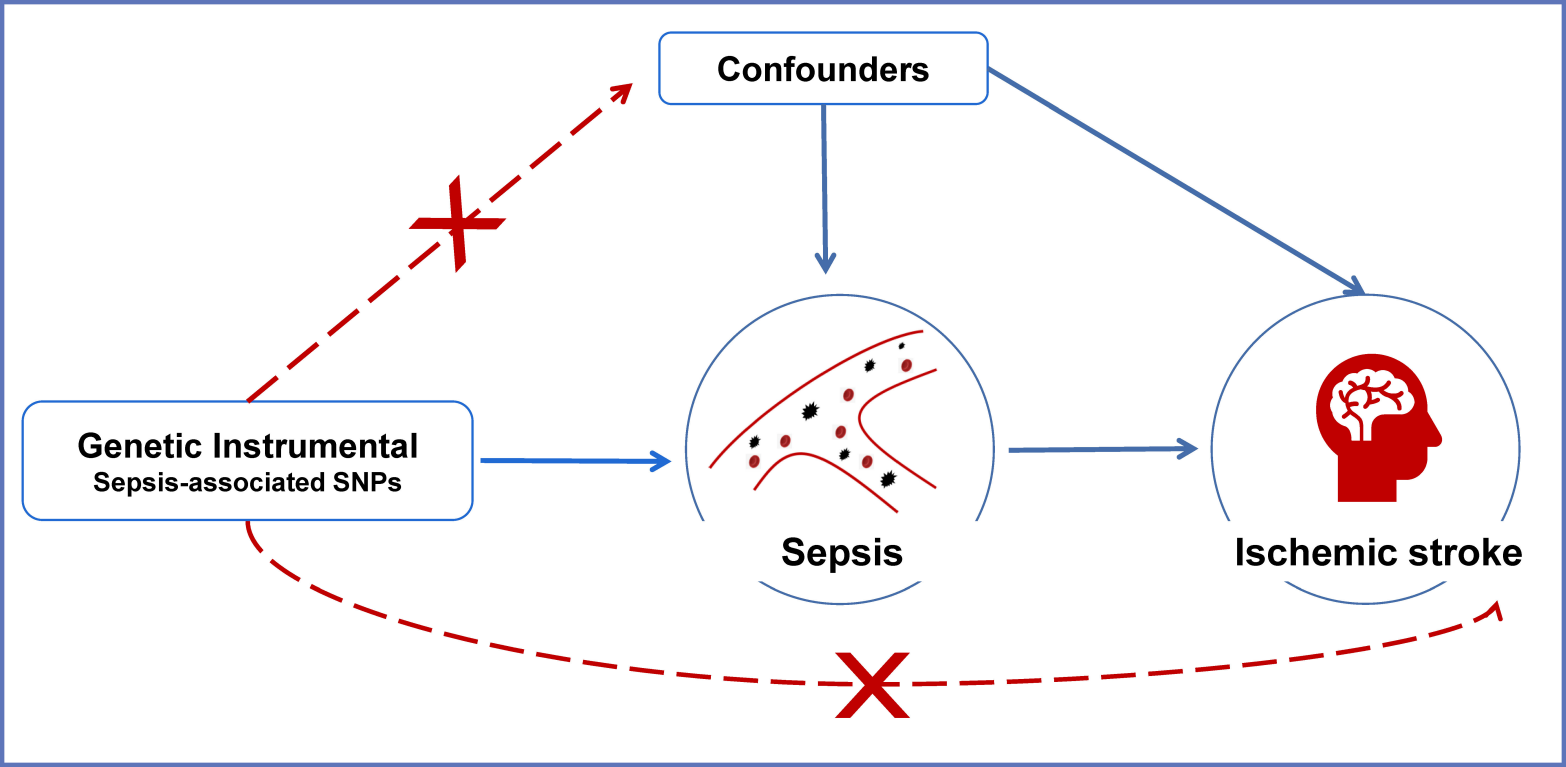
An overview of the MR assumptions. Conceptual schematic of the two-sample Mendelian randomization for the association between sepsis and the risks of ischemic stroke. SNPs indicate single-nucleotide polymorphisms.

The overview of the current Mendelian randomization (MR) study is illustrated in **Figure 1**. We used a large-scale GWAS for sepsis to obtain genetic instruments. The summary data of ischemic stroke came from a GWAS meta-analysis. The causal relationships between sepsis and ischemic stroke risk as well as the etiologic subtypes of ischemic stroke were explored by a two-sample MR analysis.

**Figure 2:**
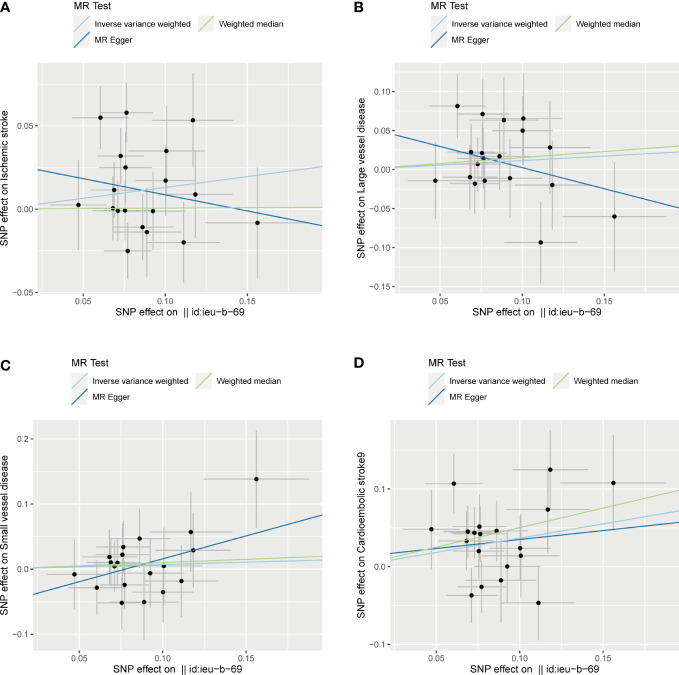
Scatter plots of the SNP effects on the sepsis and ischemic stroke risk **(A)**, ischemic stroke; **(B)**, large vessel disease;**(C)**, cardioembolic stroke; **(D)**, small vessel disease). Regression lines represent the causal effect of the sepsis on ischemic stroke risk as well as its subtypes using IVW, MR-Egger, and weighted median to estimate. Id:ieu-b-69 in the horizontal coordinate indicates sepsis.

The associations between exposure (sepsis) and outcome (ischemic stroke) were calculated with two-sample MR analysis using inverse variance weighted (IVW), weighted median, and MR-Egger regression to clarify the causal associations. The results were shown as OR and 95% CIs. The results of IVW were similar to the primary causal effect estimates and were considered consistent across all MR methods. Moreover, we performed a heterogeneity test to examine the reliability of MR estimates. Egger regression intercept was also used to estimate the magnitude of horizontal pleiotropy, which can further examine whether SNPs influence stroke risks through sepsis. All analyses were replicated on etiologic subtypes of ischemic stroke including cardioembolic stroke, large vessel disease, and small vessel disease. To facilitate interpretation, we depicted scatter plots for sepsis and ischemic stroke as well as its subtypes (**Figure 2**).

In this study, we also used MR-PRESSO global test to further detect the reliability of the results based on instrumental SNP drawn from sepsis.

MR analysis was performed in R (version 4.0.3) with R packages “Two Sample MR” and “MR-PRESSO”. *p* value < 0.05 was thought to be statistically significant.

## Results

### Study characteristics of meta-analysis

In our initial search of electronic databases, we identified 1,163 potential articles. A meta-analysis of 13 studies published between 2007 and 2022 and involving 227,463 patients was eventually conducted after a thorough examination with inclusion and exclusion criteria (3, 14–25). Of the 13 studies, 5 were conducted in the United States (3, 17, 19, 21, 23) and 2 were conducted in China (22, 25); others were conducted in European countries (15). These studies enrolled five prospective studies (15, 16, 18, 20, 23) and eight retrospective studies (3, 14, 17, 19, 21–23, 25); six of the studies recruited less than 200 patients with sepsis (15, 16, 18, 20, 21, 23), while seven had more than 200 cases (3, 14, 17, 19, 22, 24, 25). Nine were conducted in the ICU (15, 16, 18–21, 24), and four were conducted over the entire hospital (3, 14, 17, 25). Four studies reported adjusted ORs (3, 18, 21, 23), and nine reported unadjusted ORs (14–17, 19, 20, 22, 24, 25).

### Stroke and electrolyte disorder

Patients with sepsis and stroke, compared to patients without stroke, were found to be more likely to suffer from electrolyte disorders in four studies (182,980 patients) (3, 14, 17, 25). The pooled OR of stroke was 1.79 (95% CI: 1.23–3.06; *p* < 0.05), and heterogeneity showed significance (*I* ^2^ = 93.5%, *p*_h_ < 0.001) in this result. Based on sensitivity analysis, it was found that heterogeneity primarily originated from the study performed by Walkey et al. (17). By omitting this study, the pooled OR estimates received a significant boost to 2.38 (95% CI: 1.92–2.86; *p* < 0.001), with no statistical heterogeneity found (*I* ^2^ = 0.1%, *p*_h_ = 0.426). The divergent findings of this study with significant heterogeneity are possibly due to anticoagulants being more commonly used by this population, the desensitization of search codes, or changing competing risks (17). In addition, two studies (119,386 patients) examined stroke at least once in patients with electrolyte disorder during sepsis. Each of them suggested that electrolyte disorder induced by sepsis showed remarkable relevance with stroke.

### Characteristics of SNP for analysis

**Table 1** shows the exposure and outcome data summarized from GAWS. **Supplementary Table 1** presents each SNP drawn from sepsis exposure with a *p*-value < 5 × 10^−6^, along with its *F* statistic and *R* ^2^. A total of 32 instrument SNPs for sepsis were chosen, with *F* statistics ranging from 98 to 132, revealing that it was reliable for MR estimate calculation based on these SNPs and cannot generate bias due to weak instruments.

**Table 1:** Characteristics of sepsis and ischemic stroke datasets.

Exposure	Consortium	Lead SNP*	Cases/Controls	Sample size	Population	PMID
Sepsis	UK Biobank and HUNT study	19	10,154/454,764	462,918	European	PMID: 32966752
Outcome	Data source	Etiologic subtypes	Cases/Controls	Sample size	Population	
Ischemic stroke	Meta-analysis	Total	10,307/19,326	29,633	Mixed	PMID: 26935894
		Cardioembolic stroke	1,859/19,326	21,185		
		Large vessel disease	1,817/19,326	21,143		
		Small vessel disease	1,349/1,9326	20,675		

*Genome-wide significance: p < 5 × 10^−6^.

### Causal association between sepsis and ischemic stroke

In the primary analysis, no significant evidence was discovered for the causal association between sepsis and ischemic stroke (OR, 1.14, 95% CI: 0.99–1.31 for the IVW method). The results of the weighted median and MR Egger were consistent with IVW. For the etiologic subtypes of ischemic stroke, the MR results supported the idea that sepsis increases the risk of cardioembolic stroke (OR, 1.44, 95% CI: 1.14–1.83 for the IVW method). However, no causal association was observed between sepsis and other subtypes of stroke (large vessel disease: OR, 1.12, 95% CI: 0.89–1.42 for the IVW method; small vessel disease: OR, 1.07, 95% CI: 0.84–1.37 for the IVW method).

No horizontal pleiotropy and heterogeneity were examined in overall MR results (all *p*_h_ ≥ 0.05, *P*_Egger_intercept_ ≥ 0.05, **Table 2**). These results implied the reliability and persuasiveness of the results in our analysis. **Table 2** shows the detailed results for the heterogeneity test and pleiotropy test. In addition to these compelling results, the global test of MR-PRESSO analysis suggested that no horizontal pleiotropy was found in our analysis between sepsis and ischemic stroke as well as its etiologic subtypes, and no outliers were found as well (**Supplementary Table 2**).

**Table 2:** Two-sample Mendelian randomization estimations showing the effect of sepsis on the risk of ischemic stroke.

Outcomes	Etiologic subtypes	Methods	Odds ratio (95% CI)	*p*-value	*Q*-statistics	*p*_h_	Egger intercept	*p*_intercept_
Ischemic stroke	Total	MR Egger	0.82 (0.45–1.51)	0.54	26.87	0.06	0.03	0.30
		Weighted median	1.01 (0.86–1.18)	0.94				
		Inverse variance weighted	1.14 (0.99–1.31)	0.07	28.70	0.05		
	Cardioembolic stroke	MR Egger	1.26 (0.45–3.52)	0.67	20.93	0.23	0.01	0.79
		Weighted median	1.65 (1.19–2.30)	0.003				
		Inverse variance weighted	1.44 (1.14–1.83)	0.002	21.01	0.28		
	Large vessel disease	MR Egger	0.58 (0.21–1.60)	0.31	13.61	0.69	0.06	0.21
		Weighted median	1.17 (0.84–1.61)	0.35				
		Inverse variance weighted	1.12 (0.89–1.42)	0.34	15.33	0.64		
	Small vessel disease	MR Egger	2.02 (0.70–5.82)	0.21	9.09	0.94	−0.05	0.24
		Weighted median	1.11 (0.79–1.55)	0.56				
		Inverse variance weighted	1.07 (0.84–1.37)	0.57	10.54	0.91		

## Discussion

The current MR study demonstrated unexpectedly that genetically proxied sepsis exposure was specifically causally associated with cardioembolic stroke, but not with total stroke. Meanwhile, we also failed to find significant associations between genetic exposure of sepsis and large/small vessel disease.

Cardioembolic stroke refers to stroke caused by heart disease; that is, in the process of heart contraction, the micro-thrombosis in the heart falls off, causing cerebrovascular embolism, and then the stroke event occurs. Nearly 30% of ischemic strokes are due to cardioembolic stroke. Cardioembolic thrombosis-driven stroke shows more severity in clinical manifestations and late prognosis, compared with atherothrombosis. AF is considered the major cause of cardioembolic stroke (12, 13) and may increase the risk of ischemic stroke fivefold (15). Due to subclinical characteristics, patients admitted with cardioembolic stroke often receive an initial diagnosis of AF (14, 16). Previous studies indicated that 6%–20% of patients with sepsis develop new-onset AF, which is associated with higher mortality and extended hospitalization (17, 18). New guidelines for stroke prevention by the European Society of Cardiology (ESC) claimed new-onset AF in the context of sepsis as a critical yet unresolved clinical dilemma (19). Among hospitalized stroke patients, those with sepsis demonstrated a much higher risk (sixfold to be precise) of developing AF (3, 18). The prevalence of AF caused by sepsis appeared to be 10%–26% and climbed to 40% in sepsis shock (16, 20). Infections driven from the lung, abdomen, and urinary tract were deemed to be closely related to AF (3, 21). Elderly patients tended to be more susceptible to sepsis-induced AF, especially those with other cardiovascular issues (22). In a study of patients admitted to the ICU (20), Klouwenberg et al. found that AF could occur within 3 days after admission, of which the median duration was 5 h [interquartile range (IQR), 2–11], compared with 4 h (IQR, 2–10) for a recurrent episode (*p* = 0.001) (20). However, current studies on sepsis and stroke are mostly retrospective cohort studies; confounders remain to be overcome. With these lines of evidence, finding out the genetic association between sepsis exposure and stroke shows its importance. Our outcome revealed the unique association of sepsis with cardioembolic stroke, providing more aggressive stroke prevention strategies.

Sepsis is a life-threatening clinical syndrome characterized by organ dysfunction, inflammation, and electrolyte disorder caused by a patient’s dysregulated response to infection (23, 24). Although septic shock, as a typical form of distributive shock in which cardiac output is elevated or normal, manifests hypotension results from vasodilatation and organ dysfunction with a contribution from the maldistribution of blood flow, the study of myocardial dysfunction in sepsis was fraught with puzzling and contradicting findings (24). It remains to be determined whether new-onset AF is a signal of organ dysfunction and whether AF drives organ dysfunction alone, but what we do acknowledge is left ventricular (LV) dysfunction driven by sepsis-induced cardiomyopathy is common in AF patients in context with sepsis, which has a malignant effect on tissue perfusion (25, 26). Furthermore, other organ dysfunctions, especially respiratory and renal failure, are also highly correlated with AF (27, 28). Left ventricular dysfunction may also affect the function of the right heart. Right ventricular (RV) dysfunction has been reported in sepsis as well (20, 29); worse prognosis was found in heart failure patients with biventricular dysfunction (19, 30). However, isolated right ventricular dysfunction is generally not part of the definition of sepsis-induced myocardial dysfunction (24), and the relationship between right ventricular dysfunction and sepsis-induced AF warrants a more thorough investigation.

Sepsis is caused by a dysregulated host response to infection in nature (23, 24). The dysregulation in sepsis induces inflammatory factors: pathogen-associated molecular pattern (PAMP) and damage-associated molecular pattern (DAMP) upregulation and pattern-recognition receptor activation, involving a series of inflammatory responses (29). Several clinical studies have also validated the relationship between inflammation and AF in the real world. Meirehenrich et al. revealed a significant increase in C-reactive protein (CRP) levels prior to the onset of sepsis-induced AF (16). Compared with those who developed sepsis-induced AF, patients who did not have AF showed a lower CRP level (20).

Derangements in serum electrolytes are common in sepsis patients and are associated with adverse outcomes (24, 31, 32). Electrolyte imbalances such as disturbances in sodium, potassium, and magnesium contribute to a variety of cardiac arrhythmias. Lower levels of sodium and potassium contribute to the development of AF, by inducing sinoatrial node dysfunction and pulmonary vein depolarization (25, 33, 34). Magnesium modulates potassium and calcium channels in cardiomyocytes (4). Hypomagnesemia can lower the threshold for cardiac arrhythmia including AF, supraventricular tachycardia, and ventricular fibrillation (35, 36). Misialek et al. (37) suggested that there are associations between hypomagnesaemia and AF, and the severity of hypomagnesaemia is closely correlated to the rate of AF onset (38).

One strength of the current study was that the results of MR analysis reflected sepsis and had direct genetic associations with ischemic stroke (though not universally) but not with cardioembolic stroke. Previous GWAS have revealed the associations between ischemic stroke and its etiologic subtypes. Diverse loci were found to be associated with stroke subtypes, which showed unique vision into stroke mechanisms (39). Likewise, the noticeable null results of large and small vessel disease in our study suggested that sepsis affects stroke incidents through specific etiologic subtypes. More crucially, the MR method did not require direct exposure to sepsis, and it could be used any time without time or resource constraints similar to randomized controlled trials (RCTs), so as to prevent unnecessary risks and harm from being incurred by subjects (40).

However, we also have identified the following limitations in this study. First, several subgroups possess genetic variations associated with sepsis, including those that are not selected, especially those with poorer health conditions or other accompanying diseases. Moreover, invasive medical manipulation of stroke patients itself may induce infection. Second, the lack of relevant data prevented us from performing the next step on sepsis subtype analysis. Hence, further sepsis subtype analysis remains to be performed to determine the correlation between various subtypes of sepsis and cardioembolic stroke. Third, it is possible that the effect size of the relationship between sepsis and ischemic stroke could be too small to be identified, even though there was no evidence of a causal association between these two conditions. Finally, it is unfortunate that because Asian populations lacked comparable data, genetic variants were primarily derived from samples from Europe.

## Conclusion

In summary, we have strong evidence of a causal relationship between sepsis and an increased risk of cardioembolic stroke. Our study provided information that although numerous studies argue that sepsis increases the risk of stroke incidence and mortality, cardioembolic stroke should be more emphasized. Cardiogenic disease interference in due course could help sepsis patients get more benefits in stroke prevention.

## Data availability statement

The original contributions presented in the study are included in the article/**Supplementary Material**. Further inquiries can be directed to the corresponding authors.

## Ethics statement

This study is a secondary analysis conducted through existing GWAS data and UK biobank. The specific ethics and consent statements reviewed in this study can be accessed in the original publication. The patients/participants provided their written informed consent to participate in this study.

## Author contributions

All authors participated in the data collection. YML drafted the manuscript. YL, JW, PD, WW, and JJW analyzed the data. WJW and CW designed the study. YML and YLL obtained the funding. All authors contributed to the article and approved the submitted version.
