# Supplementary material for: Sepsis as an independent risk factor in atrial fibrillation and cardioembolic stroke
Source: Front Endocrinol (Lausanne). 2023 Jan 30;14:1056274. doi: 10.3389/fendo.2023.1056274 (PMC9922695; doi:10.3389/fendo.2023.1056274)
Supplement: Supplementary file 1 [file Table_1.docx]

Supplementary Table 1. Instrument SNPs selected from sepsis.

| Chr | SNP | A1 ^†^ | A2 | beta | se | P-value | R^2^ | F |
| --- | --- | --- | --- | --- | --- | --- | --- | --- |
| 2 | rs34613444 | G | T | -0.149106 | 0.0303123 | 3.50E-07 | 0.007487255 | 109.121336 |
| 2 | rs9287883 | A | G | -0.0711087 | 0.0153409 | 3.40E-07 | 0.007495324 | 109.2398172 |
| 2 | rs6435667 | G | T | -0.156173 | 0.0316103 | 7.30E-07 | 0.007279877 | 106.0767858 |
| 3 | rs4473567 | C | G | 0.100676 | 0.0231577 | 2.70E-06 | 0.0068966 | 100.4531828 |
| 3 | rs78081797 | T | C | 0.14461 | 0.0319317 | 4.20E-07 | 0.007436325 | 108.3735068 |
| 3 | rs1874981 | T | C | 0.111235 | 0.0220711 | 9.60E-07 | 0.007201188 | 104.921865 |
| 3 | rs815452 | C | T | -0.0680943 | 0.0164405 | 4.50E-06 | 0.006741445 | 98.17791509 |
| 3 | rs12639198 | A | G | 0.0758272 | 0.0160451 | 4.40E-07 | 0.00742328 | 108.1819748 |
| 4 | rs6851685 | T | G | -0.0605124 | 0.0172352 | 5.80E-07 | 0.007345351 | 107.0378747 |
| 5 | rs138181752 | A | G | 0.237458 | 0.0512906 | 4.10E-06 | 0.006769965 | 98.59609512 |
| 6 | rs375309052 | A | G | -0.0866156 | 0.0195861 | 8.00E-07 | 0.007253656 | 105.6919239 |
| 6 | rs3130320 | C | T | -0.0771445 | 0.0145878 | 8.30E-10 | 0.009022659 | 131.702486 |
| 6 | rs12203592 | T | C | 0.0470769 | 0.0168804 | 3.90E-09 | 0.008654233 | 126.277668 |
| 7 | rs144200839 | C | A | -0.090979 | 0.0208237 | 3.30E-06 | 0.006836038 | 99.56498957 |
| 8 | rs748154 | A | G | 0.0754683 | 0.015242 | 3.00E-07 | 0.007530068 | 109.7500339 |
| 8 | rs117830939 | T | C | 0.188806 | 0.0337571 | 8.50E-09 | 0.008462962 | 123.462933 |
| 9 | rs552335302 | A | C | 0.411962 | 0.0863406 | 1.30E-06 | 0.00711314 | 103.6298095 |
| 10 | rs6586161 | A | T | 0.0925214 | 0.0198718 | 5.30E-07 | 0.007370864 | 107.4124214 |
| 11 | rs722266 | C | T | -0.100221 | 0.0184403 | 7.80E-08 | 0.007894904 | 115.1098016 |
| 11 | rs11236113 | A | G | 0.116854 | 0.0248805 | 1.40E-06 | 0.007091459 | 103.3116921 |
| 11 | rs10790976 | T | C | 0.118333 | 0.0226516 | 1.50E-07 | 0.007719816 | 112.5371085 |
| 11 | rs2403552 | T | C | -0.0837135 | 0.0180548 | 4.20E-06 | 0.006762589 | 98.48794718 |
| 11 | rs4944478 | T | C | 0.0861677 | 0.0187635 | 2.60E-06 | 0.006907935 | 100.619436 |
| 12 | rs10773497 | C | T | 0.0728594 | 0.0142395 | 4.00E-07 | 0.007449987 | 108.5741037 |
| 15 | rs380804 | A | G | -0.16512 | 0.0361719 | 2.00E-06 | 0.006986262 | 101.7683564 |
| 16 | rs117613309 | C | T | 0.171087 | 0.0406606 | 9.30E-07 | 0.007210351 | 105.0563452 |
| 17 | rs4968135 | A | T | -0.0887961 | 0.0210754 | 3.10E-06 | 0.006854958 | 99.84245494 |
| 17 | rs62063644 | G | C | -0.202076 | 0.044919 | 3.60E-06 | 0.006809622 | 99.17761349 |
| 18 | rs4987768 | A | C | 0.0763707 | 0.0162731 | 4.60E-06 | 0.006734692 | 98.07890051 |
| 18 | rs79869399 | A | G | 0.315272 | 0.0689822 | 1.40E-06 | 0.007091459 | 103.3116921 |
| 19 | rs145090889 | T | C | 0.317136 | 0.0740723 | 5.60E-07 | 0.007355292 | 107.1838149 |
| 21 | rs2837606 | C | T | 0.068883 | 0.0145497 | 1.20E-06 | 0.007136481 | 103.972308 |

Chr, chromosome; SNP, single-nucleotide polymorphism.

^†^A1 is the effect allele for each SNP in the association analysis.

Supplementary Table 2. Application of MR-PRESSO to detect horizontal pleiotropy in Mendelian randomization analysis.

| **Outcomes** | **Etiologic subtypes** | **Outliers** | **Global Test-P value** |
| --- | --- | --- | --- |
| Ischemic stroke | Total | NA | 0.31 |
|  | Cardioembolic stroke | NA | 0.33 |
|  | Large vessel disease | NA | 0.32 |
|  | Small vessel disease | NA | 0.33 |
